# Spatial and temporal distances in a virtual global world: Lessons from the COVID-19 pandemic

**DOI:** 10.1057/s41267-022-00585-9

**Published:** 2022-12-31

**Authors:** Lilac Nachum, Peter J. Buckley

**Affiliations:** 1 Baruch College, City University New York, 55 Lexington Avenue, New York, NY 10010-5585 USA; 2 Leeds University Business School, University of Leeds, Leeds, LS2 9JT UK

**Keywords:** theory of FDI and the MNE (ownership-location-internalization), time orientation, geographic distance, COVID-19, teams and teamwork, foreign direct investment policy

## Abstract

The experience of COVID-19 prompted us to rethink the imperatives of distance for the organization of value-creating activities globally. We advance a conceptualization of distance as representing separation in both space and time and posit that these distance dimensions represent different kinds of separation and require varied theoretical attention. We delineate the intrinsic qualities of spatial and temporal distances and theorize the impact of this extended conceptualization of distance on major tenets of international business theory and their predictions regarding the patterns of international business activity. We illustrate the ways by which varying configurations of spatial and temporal distances serve different value-creating activities and draw their implications for countries’ global integration. We advance a call for more attention to time and temporal distance and their impact on the ways firms organize their value-creating activities in an increasingly virtual world.

## Résumé

L'expérience de COVID-19 nous a incités à repenser les impératifs de la distance pour l'organisation d'activités créatrices de valeur au niveau mondial. Nous conceptualisons la distance comme un construit représentant la séparation à la fois dans l'espace et dans le temps, et postulons que ces dimensions de la distance représentent différents types de séparation et nécessitent une attention théorique variée. Nous spécifions les qualités intrinsèques des distances temporelles et spatiales, et théorisons l'impact de cette conceptualisation étendue de la distance sur les principaux principes de la théorie des affaires internationales et leurs prédictions en matière de configurations d'activité en commerce international. Nous illustrons les façons dont diverses configurations de distances spatiales et temporelles servent différentes activités créatrices de valeur, et élaborons les implications pour l'intégration mondiale des pays. Nous appelons à accorder davantage d’attention au temps et à la distance temporelle, ainsi qu’à leur impact sur la manière dont les entreprises organisent leurs activités créatrices de valeur dans un monde de plus en plus virtuel.

## Resumen

La experiencia de COVID-19 nos instó repensar los imperativos de la distancia para la organización de actividades de creación de valor a nivel mundial. Avanzamos una conceptualización de la distancia como representación de la separación tanto en el espacio como en el tiempo y planteamos que estas dimensiones de la distancia representan diferentes tipos de separación y requieren una atención teórica variada. Delineamos las cualidades intrínsecas de las distancias espaciales y temporales y teorizamos el impacto de esta conceptualización ampliada de la distancia en los principales postulados de la teoría de los negocios internacionales y sus predicciones en relación con los patrones de la actividad de negocios internacionales. Ilustramos el modo en que las distintas configuraciones de las distancias espaciales y temporales sirven para diferentes actividades de creación de valor y extraemos sus implicaciones para la integración global de los países. Hacemos un llamamiento para que se preste más atención a la distancia temporal y al tiempo y a su impacto en la forma en que las empresas organizan sus actividades de creación de valor en un mundo cada vez más virtual.

## Resumo

A experiência do COVID-19 nos levou a repensar os imperativos de distância para a organização de atividades de criação de valor globalmente. Avançamos uma conceituação de distância como representação de separação tanto em espaço quanto em tempo e postulamos que essas dimensões de distância representam diferentes tipos de separação e requerem atenção teórica distinta. Delineamos as qualidades intrínsecas das distâncias espaciais e temporais e teorizamos o impacto dessa conceituação estendida de distância nos principais pilares da teoria em negócios internacionais e suas previsões a respeito dos padrões de atividade em negócios internacionais. Ilustramos formas pelas quais distintas configurações de distâncias espaciais e temporais atendem a diferentes atividades de criação de valor e descrevemos suas implicações para a integração global de países. Propomos um apelo por maior atenção a tempo e distância temporal e seu impacto nas maneiras pelas quais empresas organizam suas atividades de criação de valor em um mundo cada vez mais virtual.

## Abstract (zh)

COVID-19的经历促使我们重新思考距离对组织全球价值创造活动的必要性。我们将距离概念化为代表空间和时间上的分离, 并假定这些距离维度代表不同类型的分离, 并需要不同的理论关注。我们描述了空间和时间距离的内在特性, 并将这种扩展的距离概念化对国际商务理论主要原则及其对国际商务活动模式预测的影响理论化。我们说明了空间和时间距离的不同配置如何服务于不同的价值创造活动, 并讨论了它们对国家全球一体化的启示。我们呼吁更多地关注时间和时间距离及其对企业在日益虚拟的世界中组织价值创造活动的方式的影响。

## INTRODUCTION

As a theory dedicated to the understanding of business activity whose essence is value creation across space, distance has been a central construct in international business theory [see Beugelsdijk et al. (2020) for a comprehensive review]. In these discussions, distance is theorized as a multi-dimensional construct with geographic and metaphorical meanings and is maintained to exercise a strong impact on the intensity and patterns of international business activity (Alcácer, Kogut, Thomas, & Yeung, 2017; Berry, Guillen, & Zhou, 2010; Ghemawat, 2001; Johansson & Vahlne, 1977; Shenkar, 2012; Zaheer, Schomaker, & Nachum, 2012).

International business activity, however, traverses both spatial and temporal distances, but this has not been reflected in scholarly attention. While there has been a voluminous body of research on spatial distance, temporal distance has received scant attention (see Chauvin, Choudhury, & Fang, 2021; Gooris, & Peeters, 2014; Yang, Wen, Volk, & Lu, 2022; Zaheer, 1995 for notable exceptions). At best, it has been added as a control variable, and more typically it was ignored altogether. Keyword search of papers published in the *Journal of International Business Studies* yielded 571 hits for geographic distance and 166 hits for time zone.^1^ Berry et al.’s (2010) influential paper lists nine dimensions of distance relevant for MNEs, but temporal distance, conspicuously, is not among them. Theoretical development of temporal distance has thus lagged that of spatial distance, and major theoretical constructs associated with temporal location and distance have been underexplored and are poorly understood.

This attention misallocation is disturbing in an academic field whose *raison d'être* is the study of the separation of business activity in time and space. It is also inconsistent with the nature of international business activity. The intangible assets that drive this activity are assumed to be transferable over spatial distance at no cost (Dunning & Lundan, 2008). Transferability across temporal distance, in contrast, is mired with challenges, and is costly to execute. The creation and utilization of many of the intangible assets that drive international business require face-to-face human interaction and cannot be separated in time in both the production and the consumption. The shift toward coordination-intensive forms of production among firms located in different time zones has further increased the time sensitivity of international activity (Hummels, & Schaur, 2013; Yang, et al., 2022).

Studies of the two distance dimensions show that time separation has a stronger impact on a variety of outcomes of interest in international business theory than spatial distance, including collaborative knowledge creation (Espinosa, Cummings, & Pickering, 2012), productivity (Espinosa, et al., 2012; Hinds & Kiesler, 2002), MNE governance choices (Gooris & Peeters, 2014), and resource transfer among MNE subunits (Chen, & Lin, 2019; Stein & Daude, 2007).

The neglect of temporal distance undermines not only the ability to understand the implications of temporal distance but that of spatial distance as well. Many consequences assumed to spatial distance are in fact a result of temporal ones. The ignorance of temporal distance may inflate the effect of spatial separation because of omitted variables bias. This approach reflects an implicit or explicit assumption that temporal distance has no impact, an assumption that is inconsistent with research that documents the high cost of transfer and communication among entities separated in time (Chauvin, et al., 2020; Hinds et al., 2002; Hummels et al., 2013).

Studies show that when a time-zone measure, or some proxy for its consequences, are added to gravity models of business activity the impact of spatial distance drops significantly, and often turns insignificant (Espinosa, et al., 2012; Portes & Ray, 2005; Stein & Daude, 2007). Bahar (2020) found that the negative impact of spatial distance on knowledge transfer between headquarters and affiliates is significantly weakened as the temporal distance between them diminishes. The effect of one additional hour of time overlap among subunits is equivalent to a reduction of about 200 km of spatial separation between them. These findings suggest that the two distance dimensions are interdependent such that the same spatial distance affects firms differentially across different scales of temporal distance, further accentuating the need to account for temporal distance.

All this mattered less in the pre-COVID-19 era because traveling - a mode of crossing distance that lumps temporal and spatial distances together and obscures many of the differences between them - was the major means of crossing distance (Boeh & Beamish, 2012). Travel restrictions imposed by COVID-19 led to virtualization of economic activity and separation of value creation from physical location to an extent never experienced before (Cote, Estrin, Meyer, & Shapiro, 2020). This revealed the stark differences between spatial and temporal distances, as the virtualization of economic activity rendered spatial distance less relevant, but it has severe limitations in relation to temporal distance. These developments signify major shifts for international business activity and call for rethinking of the role of distance in international business theory, and its impact on the organization of value-creating activities on a global level.

In this paper, we seek to begin filling in this need and offer fresh thinking into the ways by which the increasing virtualization of business activity changes the implications of distance for international business theory and practice. Towards this end, we conceptualize distance as a construct that represents separation in both space and time, which together shape outcomes. Blending insights of global teams theories (Chauvin, et al., 2020; Mell, Jang, & Chai, 2020; Salas, Ramon, & Passmore, 2017), time economics (Bahar, 2020; Stein & Daude, 2007; Zaheer, 2000), and economic geography theories (Peuquet, 1994; Shekhar, & Xiong, 2008), we articulate the distinctive properties of temporal and spatial distances and reason that although at time they move in tandem, they are conceptually distinct (Espinosa, et al., 2012), and affect organizational outcomes differently (Chen & Lin, 2019; Gooris & Peeters, 2014), calling for different theorization so that their distinct consequences can be better understood. We conclude by extending a call for adopting a temporal lens towards international business theory and developing a research agenda around time and temporal distance in international business.

Our contribution assumes considerable importance as the virtualization of economic activity has accelerated the spread of MNE activities over space and time and led to experimentation with novel models for taking advantage of the new ways of organizing value-creating activities (e.g., “work from anywhere”). Moreover, the choices that MNEs make in re-configuring the spatial and temporal separations of their activities affect not only themselves but economies and societies as well, shaping countries’ comparative advantage and global competitiveness (Baldwin, 2019; Brakman, Garretsen, & van Witteloostuijn, 2021; Zaheer, 1995), further enhancing the importance of our contribution.

## SPATIAL AND TEMPORAL DISTANCE PROPERTIES

Spatial and temporal distances differ from each other in ways that affect their impact on international business activity in important ways. At the most basic level, these distance dimensions are both continuous and cyclical, but they differ in the scale of their cyclicality, whether around the globe or around the sun. Spatial cyclicality – referred to as Earth's circumference – is the distance around the earth (slightly over 40,000 km when measured around the equator). Temporal cyclicality revolves around the sun, in a patterned 24-h rhythm that repeats itself in a daily cycle (circa diem = “about one day”) (Shekhar, & Xiong, 2008).

These differences have important implications for business activity that takes place

across distance (Pittendrigh, 1993). Temporal cyclicality is aligned with the natural rhythm of humans, whereas spatial cyclicality is not related to it. The natural process of human life evolves in a Circadian rhythm that regulates the sleep–wake cycle and repeats itself every 24 h. A variety of human indicators are affected by time, as is vividly apparent by difficulties of adjustment to changes in time zones (Jehue, Street, & Huizenga, 1993; Lemmer et al., 2002). Managers reported a 50% drop in productivity caused by traveling and adjustment to new time zones upon arrival (Boeh & Beamish, 2012). This rhythm of human beings shapes the consequences of temporal distance for business as well. Zaheer (1995) describes how the human cycle of a day dictates market dynamics in the global foreign exchange industry and obstructs the emergence of a truly global market during a 24-h global trading cycle. No equivalent effect is caused by spatial distance, whose dynamics can be thought of as exogenous to human rhythms.

These differences in the cyclicality of the distance dimensions entail that their impact differs at different scales (Espinosa & Carmel, 2004; Zaheer, Albert, & Zaheer, 1999). The impact of spatial distance increases linearly with an increase in distance, albeit at diminishing returns. The quality and frequency of communication drops significantly at a very small increase in spatial distance, but once it reaches certain levels, an additional increase in the magnitude of spatial distance has minimal additional effect (Allen, 1977; Waber et al., 2014). Traveling costs and time exhibit a more moderate and consistent rate of diminishing returns as distance increases (Boeh & Beamish, 2012). Scale matters a great deal in relation to temporal distance as well, but its impact manifests differently (Peuquet, 1994; Zaheer et al., 1999). The sensitivity of temporal distance to the Circadian circle of human beings implies that its impact on business depends not only on the length of the distance but also on the time of the day in which activity takes place.

Of notable importance for the sake of interaction over distance is the time-overlap among the parties for the exchange, as it determines the feasibility of synchronic communication, a critical determinant of communication quality and effectiveness (Bahar, 2020; Hinds & Kiesler, 2002; Stein & Daude, 2007). Even a 1-h time difference impedes the effectiveness of communication and results in reduced productivity (Espinosa, et al., 2012; Salas, et al., 2017). Changes of 1 h associated with daylight savings were shown to have a strong impact on the communication among MNE sub-units scattered across different time zones (Chauvin, et al., 2020). Yang, et al. (2022) find that work time overlap between parents–subsidiaries reduces expatriate employment because it enables synchronic online communication to replace physical presence in subsidiaries’ foreign countries. These differences between spatial and temporal dimensions imply that their elasticities in relation to each other vary across different scales of distance (Hummels & Schaur, 2013).

Yet another difference between the two distance dimensions is that spatial separation is symmetric, that is, distance (A,B) = distance (B,A) (see Zaheer, et al., 2012 for a nuanced view of this symmetry), but separation by temporal distance is not (Espinosa & Carmel, 2004; Zaheer, 1995). Temporal separation between A and B implies that A’s time zone is different from that of B. This means that A and B would be at different points in their respective Circadian cycles at the time of the interaction, with corresponding implications for their alertness and productivity.

In addition, spatial and temporal distances are affected differently by the cardinal direction of movement across distance (Gooris & Peeters, 2014). Temporal distance changes only between East and West, whereas spatial distance changes in all cardinals, whether East/West or North/South. These differences entail that East/West move is affected by both spatial and temporal distances whereas North/South move is subject to impact of spatial distance only. This implies that movement across spatial distance may or may not be associated with change in temporal distance, but temporal distance is always associated with spatial distance (Boeh & Beamish, 2012; Jehue, et al., 1993).

Moreover, the directionality of movement, whether Eastward or Westward, matters in relation to both distance dimensions but for different reasons. The speed of humans’ adjustment to different time zones varies considerably by the direction of movement. Travel adjustment Eastward is almost 50% longer than Westward adjustment (Lemmer, et al., 2002; Waterhouse, Reilly, Atkinson, & Edwards, 2007). Kamstra, Kramer, & Levi (2000) found significant differences in the impact of time change on equity returns between the fall and spring seasons, corresponding to movement of daylight-saving Eastward or Southwards. Directionality of move between cardinals affects spatial distance as well, but this effect originates in natural attributes such as land features, e.g., uphill or downhills mountains, or winds aloft, which affect the speed of movement in different directions by land, sea, and air (Peuquet, 1994).

Further, the distance dimensions vary also in terms of the means available to bridge over them. Spatial distance can be crossed via both traveling and virtual (synchronous or asynchronous) interaction, whereas the only way to cross temporal distance is via travel. These two means of crossing distance vary in their effectiveness and are associated with different mixes of costs and benefits. They enable different amounts of human interaction and affect its quality and outcomes (Hinds & Kiesler, 2002), as was apparent during COVID-19 in the vast variations in the impact of travel restrictions and isolation across industries (Côté, et al., 2020).

Last, and by no means least, are differences in the cultural connotations of space and time and their consequences for perceptions of spatial and temporal separation across countries and cultures. Time, and temporal distance, carry strong cultural connotations (Levine, 1997; Rooney, 2012), with deep roots in countries’ histories and trajectories of economic development (Galor & Ozak, 2016). Different perceptions of time across countries were shown to have a strong impact on collaborative relationships among spatially separated teams (Saunders et al., 2004), as well as on governance choices and their outcomes (Peeters, Dehon, & Garcia-Prieto, 2015). No similar effects appear to exist in relation to space (Devine-Wright, & Clayton, 2010). Table 1 presents a summary of the qualities of spatial and temporal distances and highlights the differences between them.

**Table 1:** Distance dimensions and their distinctive properties

	Spatial distance	Temporal distance
Cyclicality	Around the globe (Earth's circumference)	Around the sun (24-h circle)
Relation to humans	No. Exogenous to humans	Yes. Identical to human endogenous clock
Scale of impact	Continuous, linear with diminishing returns	Punctuated by Circadian rhythms
Elasticity in relation to each other	Spatial distance may not be associated with crossing temporal distance	Temporal distance always associated with crossing spatial distance
Symmetry of separation	Yes. Distance(A,B) = Distance(B,A)	No. A’s time zone ≠ B’s time zone; different stages in A,B circadian circles
Cardinal direction of distance	East/West; North/South	East/West
Sensitivity to movement between cardinals	Yes. Impact of natural features (climate, wind aloft, land formation)	Yes. Impact of humans’ adjustment to different Circadian circles
Means of crossing	Travel; virtual communication (a/synchronic)	Travel
Cultural sensitivity	Low/moderate	High

### Spatial and Temporal Distances Combined

**Figure 1:**
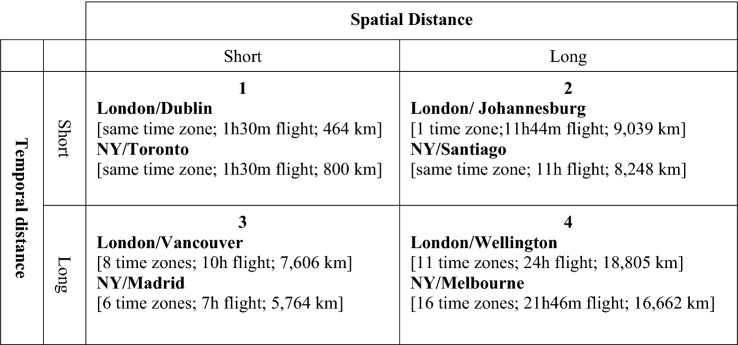
Spatial and temporal distances. Flight times for one-way, direct, shortest route. Time zone in absolute values (winter-clock time in NY and London). Time zones are drawn from imaginary lines dubbed longitudes, which separate the global into 24-hourly areas by their offsets from Coordinated Universal Time (UTC), such that each time zone is equal to a 1-h temporal distance. https://www.travelmath.com/flying-time/

Global activity evolves separation in both space and time and is thus subject to the combined effects of spatial and temporal distances, requiring a joint consideration of both distance dimensions (Chen & Lin, 2019). In Figure 1 we offer a parsimonious presentation of varying combinations of the two distance dimensions in relation to selected cities around the world, with London and New York as focal points. Temporal distance is measured by the number of time zones from London and New York (respectively, GMT and EST time zones). Spatial distance is operationalized by kilometer distance and direct flight time from these cities (flight time allows for comparability with temporal distance, as both measures are time-based). The full dataset of the distance measures is presented in “Appendix”.

As Figure 1 shows, temporal and spatial distances relate to each other in different ways, moving in tandem (Quadrants 1 and 4), where they subject firms and countries to the combined effects of separation in time and space, or departing from each other (Quadrants 2 and 3), confronting subjects with challenges of respective dimensions. The quadrants presented in Figure 1 show that the consequences of the same spatial distance vary across scales of temporal distance (the difference between quadrants 2 and 4 and between 1 and 3). Likewise, temporal distance differs in relation to different scales of spatial distance (the differences between quadrants 1 and 2 and 3 and 4) (Hummels & Schaur, 2013). In the following sections, we outline the implications of the differences between the two distance dimensions and their combined configurations for theory and practice.

## IMPLICATIONS OF A MODIFIED CONCEPTUALIZATION OF DISTANCE FOR INTERNATIONAL BUSINESS THEORY

Conceptualizing distance as a construct that combines separation in both space and time and recognizing the distinctive properties of separation along these dimensions, carries important implications for international business theory. As an organizing framework in which to present our ideas, we employ Dunning’s OLI paradigm, whose cohesive, all-embracing character makes it suitable for this purpose (Dunning & Lundan, 2008).

The OLI paradigm attributes the existence of the MNE and the patterns of its activity to three related factors, namely Ownership, Location, and Internalization advantages. Taken together, these three tenets explain why firms invest overseas and what determines the amount and composition of their international activity. We reason that these factors are modified in important ways by conceptualizing distance as encompassing separation in both space and time. We show that temporal distance affects the three dimensions of the OLI both by accentuating the impact of spatial distance, and in its own distinctive ways.

### Ownership Advantages

Ownership advantages (the O of the OLI) describe the advantages that firms possess above those of local firms that enable them to overcome liabilities of foreignness and compete successfully in foreign countries. These advantages arise either from privileged ownership of, or access to, some income-generating assets that are transferable within firms across spatial distance, and/or from the ability to coordinate these proprietary assets across countries. Dunning labeled these advantages respectively ‘Oa (asset) advantages’ and ‘Ot (transactional) advantages’ (Dunning & Lundan, 2008; Verbeke & Yuan, 2010). Explicit in these conceptualizations is the assumption that both types of assets create advantages for firms over spatial distance.

We posit that temporal distance is also likely to affect these advantages. The ability to exploit the asset-based advantages across countries (Oa) and to organize transactions among subunits separated in space (Ot) are both sensitive to temporal separation (Bahar, 2020; Boeh & Beamish, 2012). The cyclical nature of temporal distance and its punctuations by the Circadian rhythm (Table 1) open opportunities for connecting temporally separated subunits virtually. This creates time-based Oa advantages in which around-the-clock organization of work enables firms to tap into diverse sources of knowledge and expertise, utilize low-cost resources, and reduce turnaround time. In parallel, dispersion of activities across temporally separated subunits enables MNEs to appropriate greater returns from their Oa by leveraging them around the clock (Carmel, 2006; Carmel, Espinosa, & Dubinsky, 2010; Zaheer, 2000).

Temporal distance also affects the Ot advantages. In part, challenges of temporal distance to the organization of work over distance accentuate those documented extensively in relation to spatial separation (Beugelsdijk, et al., 2020; Buckley & Casson, 2020; Ghemawat, 2001), but the mechanisms that drive them differ in important ways. The management of interdependencies among subunits separated in time requires specific transaction-related capabilities that differ from those associated with the management of work across spatial distance. These capabilities need to address time-related dynamics in the workplace such as non-linearities of temporal distance as it is being punctuated by Circadian human rhythms, time-zone overlap or lack thereof, and directionality of movement across cardinals (Hinds & Kiesler, 2002) (Table 1). These capabilities could strengthen existing Ot advantages and be the source of new, time-related Ot advantages.

### Location Advantages

For international activity to take place, firms’ ownership advantages must be more profitably exploited when used with factor inputs in host countries than in the home country (Dunning, 1998). Locationally bound resources are tied to the location that gives them rise and access to them requires physical presence in this location. The distribution of these location-specific resources across countries thus shape MNE location choices such that they select countries whose resources enable them to maximize the returns on their ownership advantages (Nielsen, Asmussen, & Weatherall, 2017).

Countries differ in terms of their temporal location in relation to other countries, turning temporal location into a location characteristic that could affect location choices. This impact manifests in a variety of ways, related to temporal proximity to other countries, time overlap with those of other countries, and cardinal location (Table 1). For instance, Mumbai’s workday overlaps with countries that together account for 73% of world's GDP, making it ‘the time zone champion’. By comparison, New York’s workday overlaps with those of countries that account for only 33% of the world’s GDP (Segalla, 2010). Greater temporal overlap with other countries opens opportunities for ‘temporal brokerages’ (Mell, et al., 2020) within the MNE, which bridge subgroups with little or no temporal overlap with each other, similarly for countries' spatial position within global networks (Nachum, Zaheer, & Gross, 2008).

The magnitude of the temporal separation affects communication and control and the feasibility of synchronous communication (Chauvin, et al., 2020). These differences are related also to countries’ position along the Circadian rhythm relative to other countries, i.e., in terms of sleep time. Large temporal distance from others makes countries attractive for activities that take advantage of time-zone differences e.g., by organizing work around the clock (Marjit, 2007). Cardinal location in relation to other countries (e.g., between home and host countries) is likewise an important source of countries’ temporal advantages, as they determine exposure to spatial distance only (North/South movement) or to both spatial and temporal distances (West/East movement). These differences correspond to e.g., communication of US firms with Latin America versus Asia, and of European firms with Africa versus Russia, countries at similar spatial distances from the focal country and considerably different temporal distances.

### Internalization Advantages

The third tenet of Dunning OLI states that for foreign investment to take place it must be more beneficial for firms to internalize the use of their ownership advantages than to sell or lease their use to a third party (I advantage). Firms’ choice of internalizing cross-border operations is set where the marginal benefits of internalizing cross-border transactions are offset by the marginal cost. Spatial distance is recognized as an important determinant of these respective costs and the subsequent choices that firms make (Buckley & Casson, 1976, 2020).

Temporal separation is likely to affect these costs as well, in part raising the costs of spatial distance; in other ways exercising separate and different effects, which manifest in both markets and hierarchy, and affect their effectiveness as alternative governance mechanisms.

The cost of organizing value-creating activities hierarchically rises as temporal separation increases, particularly in activities that are intensive in information and require human interaction in real time (Stein & Daude, 2007). Increased temporal distance negatively affects the frequency and quality of communication (Chauvin, et al., 2020; Kiesler & Cummings, 2002), the amount of time it takes to accomplish work, and the quality of the output (Espinosa, et al., 2012; Hinds & Kiesler, 2002). The costs of temporal separation are particularly sensitive to time overlap, because it affects the ability to interact synchronically. Synchronous communication increases the intensity of the communication and its quality and affects the flow of knowledge and the effectiveness of collaborative work (Bahar, 2020; Espinosa, et al., 2012; Salas, et al., 2017). One study finds that a 1-h increase of temporal distance diminished synchronic communication among MNE subunits by more than 10% (but has no effect on asynchronous communication via e-mail) (Chauvin, et al., 2020).

Temporal separation also affects the costs of market transactions, as it impairs the efficiency of communication with third parties and raises the costs of establishment and maintenance of trust. This is a particular impediment in the absence of time overlaps that excludes synchronic communication. The establishment of trust relationships requires human interaction, if not in person at least virtually. This is a particular concern in transactions that are neither market nor hierarchy (Hennart, 1993), where trust substitutes for contracting and monitoring as a coordination mechanism (Alcacer, et al., 2017).

While the cost of transactions rises with temporal distance in relation to both markets and hierarchy, the rise is unlikely to be equal. The balance between these costs determines their respective advantages and is likely to vary across different activities (Buckley et al., 2020).

In Table 2 we present a summary of the impact of temporal distance on the three OLI components, in relation to those that have been theorized traditionally in relation to spatial distance.

**Table 2:** OLI framework and spatial and temporal distances

	Spatial distance	Temporal distance
Ownership	Oa – exploitation over spatial distance. Spatial mobility Ot – organizing transactions over spatial distance	Time-based Oa – around-the-clock; leverage assets across time zones Time-based Ot – organizing over temporal distance: Circadian cycle, time overlap
Location	Spatial location as location (dis)advantage; spatial proximity to other countries	Temporal location as a location dis/advantage: temporal proximity, time overlap, cardinal location (West/East; North/South) in relation to other countries
Internalization	Spatial distance as a determinant of benefit/cost of internalization across borders: Hierarchy/markets	Temporal distance as a determinant of benefit/cost of internalization across borders: communication, trust building in (a)syncronic communication

## IMPLICATIONS FOR PRACTICE

The differences between the temporal and spatial distance dimensions discussed above (Tables 1 and 2) and the varying configurations of spatial and temporal separations outlined in Figure 1 call for corresponding responses in MNEs’ organization of activities across distance and in policymaking, as we outline below.

### Implications for MNEs

Different configurations of space and time separation are suitable for different activities, reflecting variations in their sensitivity to spatial and temporal differences (Hinds & Kiesler, 2002). For instance, differences in simultaneity in the production (e.g., joint product development activities) and delivery (e.g., synchronized execution with consumers) affects the appropriate spatial and temporal configuration across different industries (Gooris & Peeters, 2014). Large temporal differences that allow for the creation of Oa temporal advantages by using around-the-clock production offer considerable potential advantage in industries in which value creation activities can be separated in time, e.g., back-office support, software development, and the likes. In contrast, Oa advantages that originate in the exploitation of temporal differences are of less value in most manufacturing industries. Such separation could even be debilitating for these industries because it challenges the potential of Ot advantages in the form of communication and coordination among subunits engaged in joint production. Such separation across time also increases the cost of transactions among MNE subunits and affects the benefits of markets versus hierarchy in serving foreign markets (Stein & Daude, 2007; Tomasik, 2013).

Likewise, different types of investment favor different configurations of separation in space and time. For horizontal, market-seeking investment, in which affiliates duplicate knowledge and business models developed at headquarters across countries, temporal proximity to headquarters and time overlaps that allow for synchronic communication could strengthen the Ot advantages. This type of investment often requires considerable human interaction with headquarters in order to administer effectively the transfer of knowledge and resources needed for affiliates to replicate HQs’ knowledge effectively in foreign countries (Bahar, 2020). In parallel, it is less (not) sensitive to spatial distance because the transfer of material goods among subunits in such investment is minimal. Temporal proximity might matter less for vertical investment, characterized by fragmented organization of production, where spatial proximity to other subunits engaged in production of complementary output could accelerate the overall speed of production and reduce the costs of transactions, enhancing the benefits of internalization.

Further, temporal separation, particularly when it is large and excludes synchronic interaction, is debilitating for the creation of Oa in knowledge-intensive industries where value creation typically requires considerable amount of real-time interaction (Carmel, 2006; Chauvin, et al., 2020; Espinosa & Carmel, 2004). In contrast, temporal separation matters less for value-added activities in which transfers are based to a greater degree on codified knowledge that can be transferred without direct human interaction (e.g., a-synchronically), rendering temporal distance less important. Bahar (2020) finds evidence that affiliates with large overlap in working hours with their headquarters are more likely to be active in knowledge-intensive industries.

Variations in the impact of temporal and spatial separation on Oa and Ot exist also in relation to different modes of international operation. Temporal separation matters for both trade and FDI, but its impact on trade is considerably smaller because the need for real-time communication is smaller among trading partners (Stein & Daude, 2007; Tomasik, 2013). In parallel, for many types of FDI spatial distance matters less, favoring different configurations of space and time separation for trade and FDI.

Temporal location is also a part of countries’ location (dis)advantages, with implications for MNE location choices and the opportunities they offer for the creation of L-advantages. Countries’ temporal location determines around-the-clock access, thus raising the opportunities for taking advantage of immobile locational resources like skills and knowledge. This is notably apparent in relation to labor, where temporal location shaped the patterns of supply and demand for labor across countries (Brakman, et al., 2021). Similar effects are apparent in relation to suppliers and local partners, with implications for local specialization and creation of global production networks by MNEs (Acemoglu & Restrepo, 2018).

### Implications for Policymakers

Countries’ ability to integrate in global production networks is determined by the combined effect of their spatial and temporal location in relation to other countries (Figure 1), calling for policies that are responsive to specific configurations of spatial and temporal location.

Historical accounts show that some policymakers have adopted an active approach to the management of time throughout history, with a view towards amending their temporal location in the service of global integration (Rooney, 2020). Setting the same time zone to a trading partner had been behind Argentina’s flip-flopping its clock during most of the 20th century between UTC-4, where its geographic location places it, and UTC-2 that its trading relationships favor. Since 1993, it has been on UTC-3. Trading partners have also sought to influence each other’s time zones, as American traders did in the 19th century when they persuaded Samoans to align their island time with that of nearby US-controlled American Samoa to make trading easier. More than a century elapsed until Samoa shifted its time to its locational time zone (Calabi, 2013; Wong, 2015).

Investment promotion policies should be extended to include temporal characteristics, as supplement to the spatial characteristics that were included in these policies throughout history (Henrikson, 2002; Nachum, Livanis, & Hong, 2021; Ward, 2005). Brazil’s branding itself as a location for collaboration-intensive software development because its time zone overlaps with primary partners in North America is a case in point. Time zone, as it enables simultaneous collaboration, has occupied central place in Brazil’s attempts to establish itself as a location for IT software and services (Prikladnicki, & Carmel, 2013). India, and to some extent the Philippines, in contrast have branded themselves as desired locations for investments based on temporal differences that allow them to take advantage of around-the-clock work (Carmel, 2006).

## DISCUSSION AND CONCLUSION

In this paper we contribute to the development of theories of distance in international business by conceptualizing distance as a construct that combines spatial and temporal dimensions (Berry et al., 2010; Ghemawat 2011; Alcácer, et al., 2017). The implications of spatial distance for resource transfer and communication among MNE sub-units have long been theorized as a prime determinant of the scope of international activity (Buckley & Casson, 1976, 2020; Dunning & Lundan, 2008). By adding the implications of temporal distance to these theorizations we offer a more coherent framework in which to theorize the patterns and intensity of international business activity and draw implications for practice.

In doing this, we contribute to a small but growing set of studies that has started to articulate the implications of temporal distance for MNEs (e.g., Bahar, 2020; Chauvin, et al., 2021; Gooris et al., 2014; Mell, et al., 2020; Yang, et al., 2022; Zaheer, 1995). Our contribution bridges the literature on spatial distance in international business with that of teamwork and the organization of work over temporal distance. Specifically, we contribute to the understanding of the relationships between the two distance dimensions and their combined and separated effects as they shape the consequences of distance for international business.

These contributions are of heightened contemporary significance. The increased virtualization of business activity entails a growing need for better understanding of the implications of spatial and temporal separations, as they affect firms, industries, and activities. We hope that the insights we offered in this paper regarding the distinct qualities of these distance dimensions and the ways they relate to each other would serve to support the development of a research agenda in which international business is treated as an activity that takes place across both spatial and temporal distances.

We also hope that this insight would guide firms as they construct the shape of their activities in this changing reality. The experience of prolonged lockdowns and traveling bans imposed by COVID-19 had equipped us with renewed insights into these issues and inspired our conceptualization of distance and its implications in this paper. We hope that these insights would feed into MNEs’ reevaluation of the appropriate configurations of spatial and temporal distances for international activities (McKinsey, 2021).

### Limitations and Future Research

Our study opens a large scope for future research to develop the ideas we advanced in this paper and address the limitations of our work. Perhaps the most immediate task for future research is to supplement our conceptual work by empirical testing. Our theory generates testable propositions regarding the impact of distance configurations on the patterns and intensity of international business activity. We also offer some tools to operationalize major theoretical constructs that could serve this research (Appendix A and Figure 1).

Additional work by future research is warranted also with reference to the relationships between temporal and spatial distances. Our discussions of these relationships, as summarized in Figure 1, presented the two distance dimensions as dichotomous and hid the richness of the nuances of the relationships along these continuous measures. Future research may address the limitations of this parsimonious approach and deepen the understanding of the way by which the scales of both distance dimensions affect outcomes (Bahar, 2020; Zaheer, et al., 1999). This would also deepen the understanding of the ways by which configurations of activities in space and time might serve as a source of MNE differentiation and competitive advantage.

Further, our theory focused predominantly on the impact of distance dimensions on internal MNE organization of work. Future research may supplement our discussions by extending them to the intra-firm context and examine the ways by which different distance configurations affect MNE relationships with third parties, via arm’s-length or relational relationships (Chen et al., 2019).

There is also a need for an on-going evaluation of the relationships between spatial and temporal distances, and the way they affect international business activity, as technology continuously modifies the cost of crossing both spatial and temporal distances. Means of crossing distance have changed considerably throughout history because of technological developments and will continue to evolve (Antras, Redding, & Rossi-Hansberg, 2020; Baldwin, 2019). These developments affect the costs and benefits of the two distance dimensions, with important implications for the issues we raised in this paper.

## NOTES

^1^Similar patterns are apparent in other leading international business journals, with 35 and 12 hits, respectively, for geographic distance and time zone in the *Journal of World Business* and 114 and 22 in the *Global Strategy Journal*. The corresponding figures in the journals of the *Academy of Management* are 146 hits for distance and 21 for time zone.

## Appendix: Temporal and spatial distances, selected cities

**Table:**

	From London			From New York		
	Time zone (number)	Flight time (hours, minutes)	Distance (km)	Time zone (number)	Flight time (hours, minutes)	Distance (km)
Addis Ababa	2	7.49	5889	7	14.27	11219
Athens	2	3.29	2394	7	10.22	7945
Austin, TX	6	10.21	7928	1	3.34	2435
Bangkok	6	12.22	9544	11	17.5	13948
Beijing	7	10.38	8160	12	14.11	11015
Buenos Aires	4	14.18	11,102	1	11.03	8492
Cairo	1	4.52	3513	6	11.44	9041
Dhaka	5	10.28	8015	10	16.16	12682
Dubai	3	7.19	5479	8	14.12	11028
Dublin	0	1.5	464	5	6.52	5130
Frankfurt	1	1.18	639	6	8.14	6219
Helsinki	2	2.46	1826	7	8.45	6636
Istanbul	2	3.37	2504	7	10.33	8088
Johannesburg	1	11.44	9039	6	16.27	12833
Kabul	3:30	7.37	5721	8:30	14	10864
Lagos	0	6.43	5005	5	7.24	5549
Los Angeles	8	11.25	8778	3	5.23	3944
Madrid	1	2.4	1264	6	7.41	5785
Malaga, Spain	1	2.35	1678	6	7.5	5907
Melbourne	9	21.3	16,898	14	21.13	16672
Mexico City	6	11.37	8943	1	4.4	3358
Moscow	2	3.37	2508	7	9.52	7531
Mumbai	4:30	9.27	7204	9:30	16.07	12564
Nairobi	2	8.57	6805	7	15.14	11849
New Delhi	4:30	8.51	6723	9:30	15.08	11777
Oslo	1	1.56	1157	6	7.52	5931
Paris	1	0.55	341	6	7.46	5851
Rabat	0	3	2016	5	7.46	5851
Riyadh	2	6.39	4955	7	13.35	10529
Rome	1	2.17	1434	6	9.05	6907
Santiago, Chile	5	14.59	11,651	0	10.43	8218
Sao Paulo	4	12.16	9470	1	10.01	7657
Seoul	8	11.32	8880	13	14.16	11077
Shanghai	7	11.59	9235	12	15.16	11882
Stockholm	1	2.17	1436	6	8.23	6337
Tel Aviv	2	4.55	3560	7	11.51	9135
Tokyo	8	12.25	9585	13	14.01	10871
Toronto	5	7.37	5731	0	1.11	551
Vancouver	8	9.57	7606	3	5.22	3915
Vienna	1	2.2	1238	6	8.58	6815
Wellington	11	23.52	18,805	16	18.23	14394
Zurich	1	1.28	778	6	8.23	6342
New York/London	5	7.27	5586	5	7.27	5586
Min	0	0.55	7.606	0	1.11	551
Max	11	23.52	18805	16	21.13	16672
Correlation with time zone, *p* values		0.79	0.71		0.57	0.58

https://www.travelmath.com/flying-time/

Flight times for one way, direct, shortest route.

Time zone in absolute values (winter-clock time in NY and London). One time zone equal 1 h.
